# Risk of Hepatitis B Virus Reactivation in Patients with Resolved Infection on Therapy with Corticosteroids and Conventional Synthesis Immunosuppressants for Kidney Disease: A Single-Center Analysis of 258 Patients

**DOI:** 10.5152/tjg.2023.22511

**Published:** 2023-10-01

**Authors:** Pingyang Han, Ziqiu Wang, Zhaohui Wang

**Affiliations:** 1 Department of Nephrology, Ruijin Hospital, Shanghai Jiao Tong University Faculty of Medicine, Shanghai, China; 2 Department of Nephrology, The Affiliated Hospital of Hangzhou Normal University, Hangzhou, Zhejiang Province, China; 3 Department of Clinical Medicine, Shanghai Jiao Tong University Faculty of Medicine, Shanghai, China

**Keywords:** Anti-HBc-positive patients, HBV reactivation, hepatitis flare, immunosuppressive therapy, kidney disease

## Abstract

**Background/Aims:**

The risk of hepatitis B virus reactivation in patients with a previously resolved hepatitis B virus infection on therapy with corticosteroids and conventional synthesis immunosuppressants for kidney disease has not been well described.

**Materials and Methods:**

We performed a retrospective study on the risk of hepatitis B virus reactivation in patients with a previously resolved hepatitis B virus infection on therapy with corticosteroids and conventional synthesis immunosuppressants for kidney disease between January 2012 and December 2021 in the Department of Nephrology at Ruijin Hospital.

**Results:**

A total of 258 patients with a previously resolved hepatitis B virus infection [all treated with high-dose corticosteroids, of whom 192 were receiving corticosteroids combined with conventional synthesis immunosuppressant therapy, including cyclophosphamide (155), cyclosporine A (14), mycophenolate mofetil (14), and tacrolimus (9)] were enrolled. During a mean follow-up time of 21.66 months (range 9-70 months), hepatitis B virus reactivation was not observed in these patients.

**Conclusions:**

Among patients with a previously resolved hepatitis B virus infection on therapy with corticosteroids and conventional synthesis immunosuppressants for kidney disease, hepatitis B virus reactivation was not common and severe, suggesting that universal prophylaxis may not be justified or cost-effective in this clinical setting.

**Main Points**

- Our retrospective study showed that in patients with resolved hepatitis B virus (HBV) infection on therapy with corticosteroids and conventional synthesis immunosuppressants for kidney disease, HBV reactivation was not observed.
- Universal prophylaxis may not be justified or cost-effective in this clinical setting.

## Introduction

In patients with chronic hepatitis B virus (HBV) infection and/or those who are inactive HBsAg carriers treated with immunosuppressants, such as rituximab or prednisone ≥20 mg/day for at least 4 weeks, HBV reactivation (HBVr), as defined by standard criteria,^1^ has been well recognized.^2,3^ Prophylactic nucleoside analog therapy has been shown to reduce the incidence of HBVr in HBsAg-positive patients and is recommended in current treatment guidelines.^4^ In patients with a previously resolved HBV (prHBV) infection (HBsAg-negative/anti-HBc-positive, undetectable serum HBV DNA), HBVr has also been reported, mainly occurring in those receiving anti-CD20 agents (e.g., rituximab) or bone marrow/hematopoietic stem cell transplantations.^5,6^ Among patients with a prHBV infection who were treated with corticosteroid monotherapy or corticosteroid in combination with conventional synthesis immunosuppressant (csIS) therapy, HBVr has also been reported, but there are limited data to classify the risk.^7^ The prevalence of a prHBV infection is very common, with a high seroprevalence of anti-HBc in East Asia (estimated between 13.5% and 40.9%).^8–10^ There is a pressing need to define the risk of HBVr during immunosuppressive therapy in these populations. The risk of HBVr in patients with a prHBV infection on corticosteroid monotherapy or corticosteroid in combination with csIS therapy for kidney disease has not been well described. Herein, we performed a retrospective study to assess the risk of HBVr in these patients.

## Materials and Methods

### Study Design and Setting

The study was a retrospective observational study. We included patients who had a prHBV infection (HBsAg-negative/anti-HBc-positive, undetectable serum HBV DNA) on therapy with corticosteroids and csISs for kidney disease in Department of Nephrology at Ruijin Hospital from January 2012 to December 2021. The international medical association code of ethics was followed in all aspects of the study protocol, including access to and use of patients’ clinical information. The study was approved by the Ruijin Hospital Ethics Committee at the Shanghai Jiao Tong University Faculty of Medicine (2010 No.29).

### Study Population

The inclusion criteria of the patients were as follows: (1) patients with kidney disease who needed high-dose (prednisone or equivalent ≥20 mg/day) corticosteroid monotherapy or high-dose corticosteroid in combination with csIS therapy for at least 3 months; (2) a prHBV infection was identified by serum HBV markers [negative HBsAg (<0.05 IU/mL) and positive anti-HBc [≥1.0 S/CO (sample/cutoff)], tested by chemiluminescent immunoassays) and negative serum HBV DNA (<10 IU/mL, tested by Abbott RealTime HBV Assay, Abbott Laboratories, Des Plaines, IL, USA) before immunosuppressive therapy; and (3) monitoring of liver function monthly and/or serum HBV markers and HBV DNA every 1-3 months was performed after the initiation of immunosuppressive therapy and lasted at least 6 months after the completion of immunosuppressive therapy. Because of the different economic situation and subjective will of patients and the different personal choice of doctors, not all patients had been monitored for serum HBV markers and HBV DNA. The exclusion criteria were as follows: (1) patients with positive-HBsAg; (2) patients with concomitant chronic liver disease as a result of chronic hepatitis C and D viral infection; Wilson’s disease; autoimmune hepatitis; primary biliary cirrhosis; and a previous history of anti-HBV therapy; and (3) patients with therapies of rituximab or other biological agents. As a result, a total of 258 patients with a prHBV infection were enrolled [all of whom were anti-HBc-positive, and of whom 175 were simultaneously anti-HBs-positive (≥10.0 mIU/mL)]. All HBV markers were tested in our hospital.

### Hepatitis B Virus Reactivation

Hepatitis B virus reactivation was defined as the new appearance of HBV DNA in an individual with previously undetectable levels or demonstration of reverse seroconversion to HBsAg-positive status. Hepatitis flare is frequently determined to be present when there is at least a 2- to 3-fold elevation in alanine aminotransferase (ALT) above the patient’s baseline.^1^

### Data Collection

All patient data were collected from individual electronic patient records. This included patient demographics, diagnosis, drug regimen, serum HBV markers, HBV DNA levels, and liver function indicators of pre-, intra-, and post-treatment.

### Statistical Analysis

The Statistical Package for Social Sciences version 21.0 (IBM Corp.; Armonk, NY, USA) and Microsoft® Excel® 2010 were used for the statistical analysis. Quantitative data were analyzed by *t*-test for comparison of mean values of 2 independent samples, and qualitative data were analyzed by the chi-square test for comparison of 2 or more constituent ratios. For all tests, the 2-tailed significance level *α* was set as 0.05.

## Results

### Characteristics of Enrolled Patients

In this group of patients, the most common kidney disease was primary nephrotic syndrome (53.9%), with membranous nephropathy (MN, 34.5%) as the main pathological type, followed by IgA nephropathy (21.3%) and anti-neutrophilic cytoplasmic antibody (ANCA)-associated glomerulonephritis (14.0%). The demographic characteristics of the 258 patients are summarized in Table 1.

Overall, all patients (100%) received a high-dose corticosteroid therapy (prednisone 20-60 mg/day, mean: 42.57 mg/day) for a mean time of 6.11 months (range 3-24 months), of whom 192 patients (74.4%) received corticosteroid in combination with csIS therapy. The most commonly used csIS was cyclophosphamide (60.1%). Twenty-six patients (10.1%) received methylprednisolone pulse therapy (11 patients with 200 mg/day and 15 patients with 500 mg/day) for 3 days before routine dosages. The treatment conditions are summarized in Table 2.

### Incidence of Hepatitis B Virus Reactivation

Before receiving immunosuppressive therapy, all 258 patients were positive for serum anti-HBc, including 175 patients (67.8%) who were simultaneously positive for serum anti-HBs. All patients had undetectable (<10 IU/mL) baseline HBV DNA. The liver function of all the 258 patients (100%) was monitored monthly, and 106 of them (41.1%) also monitored serum HBV markers and HBV DNA every 1-3 months after the initiation of immunosuppressive therapy, and the monitoring time lasted at least 6 months after the completion of immunosuppressive therapy.

The total monitoring time of liver function fluctuated between 9 and 70 months (mean 17.33 months). No hepatitis flare was observed during and after the treatment. In 106 patients who were monitored for serum HBV markers and HBV DNA, the total monitoring time fluctuated between 9 and 70 months (mean 21.66 months). No negative-to-positive HBsAg reverse seroconversion and increased HBV-DNA replication levels were observed.

However, some changes with serum HBV markers in the endpoint of observation were observed in the 106 patients, including 8 patients with anti-HBc seroconversion from positive to negative, 14 patients with anti-HBs seroconversion from positive to negative, and 9 patients with anti-HBs seroconversion from negative to positive (Table 3).

There were no statistically significant differences in the clinical features and immunosuppressive therapies between patients with (group A, n = 106) and without (group B, n = 152) monitoring for serum HBV markers and HBV DNA after the treatment (Table 4).

## Discussion

In clinical practice, a variety of primary or secondary autoimmune kidney diseases, including nephrotic syndrome, IgA nephropathy, ANCA-associated glomerulonephritis, or lupus nephritis, usually require high-dose corticosteroid monotherapy or corticosteroid in combination with csIS therapies. Combination therapy with corticosteroid and monthly cyclophosphamide pulse therapy is a commonly used treatment regimen. Other csISs, including mycophenolate, cyclosporine A, and tacrolimus, are also used frequently.

In the past decade, a large number of studies have reported the occurrence of HBVr in patients receiving immunosuppressive therapy. The reactivation of hepatitis B in the context of immunosuppressive therapy may be severe and potentially fatal. Hepatitis B virus reactivation mainly occurs in HBsAg- and anti-HBc-positive patients, but it also occurs in individuals with a prHBV infection.^11^ Among patients with a prHBV infection, HBVr mainly occurred in patients receiving rituximab-containing regimens with or without hematological diseases. In contrast, patients with non-hematological diseases or receiving rituximab-free regimens, including nonbiologic or biologic disease-modifying antirheumatic drugs (DMARDs), had a low risk of HBVr and may not require anti-HBV prophylaxis.^12–14^ Some studies reported that in anti-HBc-positive patients with rheumatic diseases treated with conventional synthetic DMARDs, including methotrexate, tacrolimus, and azathiopurine, the risk of HBVr was also very low.^15,16^ Risk factors for HBVr in these patients may include negative or low titers of HBsAb, advanced age, and a history of the use of ≥ 3 classes of immunosuppressants.^14–16^

The clinical practice guidance for HBVr in patients treated with immunosuppressive therapy (IST) developed by the Asian-Pacific Association for the study of the liver (APASL) in 2021^17^ showed that the risk of HBVr is 5 to 8 times higher among those patients who are HBsAg positive as compared to those who were HbsAg negative but anti-HBc positive. Based on the type and duration of IST and the status of HBV infection, the risk of HBVr was established to be low (<1%), moderate (1%-10%), and high (>10%). In patients with a prHBV infection who received cytotoxic chemotherapy (except anthracyclines) and steroid (high dose) ≥20 mg/day, the risk of HBVr was low (<1%). While even in HBsAg-positive patients who received treatments of methotrexate, azathioprine, and steroid (low dose <10 mg/day), the risk of HBVr was also low (<1%). For those with low risk of HBVr, including HbsAg-positive and HbsAg-negative but anti-HBc-positive patients, initiation of pre-emptive nucleos(t)ide analogues (NUCs) was based on whether the patients had advanced fibrosis or cirrhosis. If the patients had advanced fibrosis or cirrhosis, pre-emptive NUCs should be initiated. While for those who had no advanced fibrosis or cirrhosis, serum ALT should be monitored every 3 months, and if elevated ALT >2 × baseline detected at monitoring, HBsAg and HBV DNA should be performed and high-resistant barrier NUCs initiated if either test positive. Our research is consistent with the literature. In this study, all 258 patients received prednisone ≥20 mg/day for at least 3 months with a mean treatment time of 6.11 months (range: 3-24 months). Of these patients, 74.4% of them received corticosteroids in combination with csIS therapy, with cyclophosphamide as the most commonly used agent, followed by mycophenolate mofetil, and calcineurin inhibitors. No hepatitis flare and HBVr was observed during a mean follow-up time of 21.66 months.

The precise mechanism by which HBVr occurs is unclear, and the initial event is thought to be a disruption in the ability of the host immune system to control HBV replication.^18^ Among HBsAg-positive patients receiving corticosteroids, HBVr has occurred both with high-dose, rapidly tapered regimens and moderate-dose, prolonged regimens.^3^ The increased viral replication may be due, in part, to a corticosteroid-responsive element in the HBV genome that stimulates viral replication and transcriptional activity.^19^

Hepatitis B virus reactivation refers to a sudden and significant increase in HBV replication (HBV DNA level), usually accompanied by an increase in serum transaminase levels. In patients with resolved infection, reactivation has been considered to occur upon the demonstration of reverse seroconversion to HBsAg-positive status.^1^ In our 258 patients, no hepatitis flare was observed in the course of regular and continuous monitoring for liver functions during and after the immunosuppressive therapy. In 106 (41.09%) of the patients who were also monitored for HBV markers and HBV DNA levels regularly, reverse seroconversion to positive HBsAg status and increased HBV-DNA replication levels were not observed. Meanwhile, there were no statistically significant differences in the clinical features and immunosuppressive therapies between patients with and without monitoring for serum HBV markers and HBV DNA levels.

There were some limitations in this study. First, it was a retrospective study, and some of the patients did not monitor serum HBV markers and HBV DNA regularly, and hence the actual incidence of HBVr may be underestimated. Second, the sample size was a limitation of the study, and since the rate of HBVr in these patients was expected to be low, a large sample size is required to make a significant estimation of the rate of HBVr. Third, there was some heterogeneity in the medication of patients in this group, which had some influence on the interpretation of the results. The actual rate of HBVr in these patients still needs some prospective, large sample size, and more homogeneous studies to confirm.

In summary, our study showed that in patients with a prHBV infection on therapy with high-dose corticosteroids (prednisone or equivalent ≥20 mg/day) in combination with/without csIS for kidney disease, HBVr and hepatitis flare were not observed, suggesting that HBVr is not common and severe in this disease entity, and the common preventive anti-HBV treatment may be unreasonable and not have economic benefits.

## Figures and Tables

**Table 1.** Demographic Characteristics of the 258 Patients with a prHBV Infection

	Number (%)
Age (mean)	18-87 (52)
Sex (female/male)	109/149
Diseases	
Nephrotic syndrome	139 (53.9)
MN	89 (34.5)
FSGS	14 (5.4)
MCD	13 (5.0)
IgAN	9 (3.5)
MPGN	8 (3.1)
Lupus nephritis	6 (2.3)
IgAN	55 (21.3)
ANCA-associated glomerulonephritis	36 (14.0)
Lupus nephritis	10 (3.9)
Primary chronic glomerulonephritis	8 (3.1)
Anti-GBM disease	5 (1.9)
Others	5 (1.9)

ANCA, anti-neutrophilic cytoplasmic antibody; FSGS, focal segmental glomerulosclerosis; GBM, glomerular basement membrane; IgAN, IgA nephropathy; MCD, minimal change nephropathy; MN, membranous nephropathy; MPGN, membranoproliferative glomerulonephritis; prHBV, previously resolved HBV.

**Table 2.** Types of Drug Treatment, Dosages, and Treatment Duration of 258 Patients

Drugs	Number, (%)	Dosage (Mean)	Treatment Duration, Months (Mean)
Prednisone (Pred)	258 (100)	20-60 (42.57) mg/day	3-24 (6.11)^a^
Cyclophosphamide (with Pred)	155 (60.1)	1.8-11.2 (4.86) g^b^	3-14 (6.87)
Cyclosporin A (with Pred)	14 (5.4)	150-250 (194.12) mg/day	3-29 (8.6)
Mycophenolate mofetil (with Pred)	14 (5.4)	0.5-1.5 (1.0) g/day	3-29 (15.4)
Tacrolimus (with Pred)	9 (3.5)	4-7 (5.25) mg/day	3-23 (11.56)

^a^Treatment duration with prednisone ≥20 mg/day; 11 and 15 patients accepted a methylprednisolone pulse therapy of 200 mg/day ×3 days and 500 mg/day ×3 days initially.

^b^Total cumulative dosages.

**Table 3.** The HBV Markers and HBV-DNA Levels Before and After Immunosuppressive Treatment in 106 Patients

	Before Treatment		After Treatment	
	Number (%)	Value (mean)	Number (%)	Value (mean)
Anti-HBs, mIU/mL				
Positive	73 (68.9)	10.4-970.0 (144.7), with 3 >1000	68 (64.2)	10.4-986.6 (126.6), with 3 >1000
Negative	33 (31.1)	<10	38 (35.8)	<10
HBsAg, IU/mL				
Negative	106 (100)	<0.05	106 (100)	<0.05
HBeAg, S/CO				
Negative	106 (100)	<1	106 (100)	<1
Anti-HBe, S/CO				
Positive	38 (35.8)	0.02~0.99 (0.51)	31 (29.2)	0.02-0.99 (0.55)
Negative	68 (64.2)	>1	75 (70.8)	>1
Anti-HBc, S/CO				
Positive	106 (100)	1.03~12.53 (5.91)	98 (89.9)	1.26-11.73 (5.52)
Negative	0	<1	8	<1
HBV-DNA, IU/mL	106 (100)	<10	106 (100)	<10

No HBVr was observed in a mean observation time of 21.66 months.

HBV, hepatitis B virus.

**Table 4.** Comparison of the Clinical Features and Immunosuppressive Therapies Between Patients With (Group A) and Without (group B) Monitoring for Serum HBV Markers and HBV DNA Levels

	Group A (n = 106)	Group B (n = 152)	*P*
*Clinical features*			
Age (mean)	25-87 (50.64)	18-84 (52.31)	.367
Sex (female/male)	40/66	69/83	.220
Disease (%)	106 (41.09)	152 (58.91)	.625
Nephrotic syndrome	62	77	/
IgAN	19	36	/
ANCA-associated GN	14	22	/
Others	11	17	/
*Therapies*			
Prednisone (Pred)			
Number (%)	106 (100%)	152 (100%)	/
Dosage (mg/day, mean)	20-60 (43.06)	20-60 (42.24)	.579
Treatment duration (months, mean)	3~24 (6.12)	3-14 (5.86)	.159
Cyclophosphamide (with Pred)			
Number (%)	67 (63.21)	88 (58.00)	.414
Dosage (mean)	1.8~10.6 (4.94)	1.8-11.2 (4.80)	.660
Treatment duration (months, mean)	3-14 (7.02)	3-14 (6.76)	.829
Others			
Number (%)	16 (15.10)	25 (16.45)	.770
Treatment duration (months, mean)	3-23 (11.44)	3-29 (8.31)	.205

ANCA, anti-neutrophilic cytoplasmic antibody; HBV, hepatitis B virus; GN, glomerulonephritis; IgAN, IgA nephropathy. Others include cyclosporin A, mycophenolate mofetil, and tacrolimus.
